# Computerized training of non-verbal reasoning and working memory in children with intellectual disability

**DOI:** 10.3389/fnhum.2012.00271

**Published:** 2012-10-02

**Authors:** Stina Söderqvist, Sissela B. Nutley, Jon Ottersen, Katja M. Grill, Torkel Klingberg

**Affiliations:** ^1^Department of Neuroscience, Karolinska InstitutetStockholm, Sweden; ^2^Stockholm Brain InstituteStockholm, Sweden; ^3^Vestre Viken Hospital Trust, Drammen Hospital, Centre of HabilitationDrammen, Norway

**Keywords:** intellectual disability, training, working memory, non-verbal reasoning

## Abstract

Children with intellectual disabilities show deficits in both reasoning ability and working memory (WM) that impact everyday functioning and academic achievement. In this study we investigated the feasibility of cognitive training for improving WM and non-verbal reasoning (NVR) ability in children with intellectual disability. Participants were randomized to a 5-week adaptive training program (intervention group) or non-adaptive version of the program (active control group). Cognitive assessments were conducted prior to and directly after training and 1 year later to examine effects of the training. Improvements during training varied largely and amount of progress during training predicted transfer to WM and comprehension of instructions, with higher training progress being associated with greater transfer improvements. The strongest predictors for training progress were found to be gender, co-morbidity, and baseline capacity on verbal WM. In particular, females without an additional diagnosis and with higher baseline performance showed greater progress. No significant effects of training were observed at the 1-year follow-up, suggesting that training should be more intense or repeated in order for effects to persist in children with intellectual disabilities. A major finding of this study is that cognitive training is feasible in this clinical sample and can help improve their cognitive performance. However, a minimum cognitive capacity or training ability seems necessary for the training to be beneficial, with some individuals showing little improvement in performance. Future studies of cognitive training should take into consideration how inter-individual differences in training progress influence transfer effects and further investigate how baseline capacities predict training outcome.

## Introduction

A now growing literature is showing that cognitive functions, such as working memory (WM), can be positively influenced by targeted and intensive training (Klingberg et al., 2005; Klingberg, 2010; Diamond and Lee, 2011; Morrison and Chein, 2011). Using computerized versions of training programs has allowed for the implementation of adaptive algorithms that ensures that the level of task difficulty is always challenging for the individual, something that has been shown to be crucial for the training to be effective (Klingberg, 2010). Such training has been shown to improve WM performance in healthy children and adults (Olesen et al., 2004; Jaeggi et al., 2008; Thorell et al., 2009; Bergman Nutley et al., 2011) and in children with attention-deficit hyperactivity disorder (ADHD) (Klingberg et al., 2002, 2005; Beck et al., 2010; Holmes et al., 2010; Mezzacappa and Buckner, 2010) children born preterm (Lohaugen et al., 2011) and adults recovering from stroke and other acquired brain injuries (Westerberg et al., 2007; Lundqvist et al., 2010). As the studies mentioned above show improvements in performance on WM tasks dissimilar to those trained on, this is assumed to reflect an increase in capacity and/or general skills rather than the development of task-specific strategies (Klingberg, 2010).

A cognitive function that is related to WM is reasoning ability (also referred to as fluid intelligence or reasoning, Gf) (Conway et al., 2003; Kane et al., 2004). Reasoning ability refers to the ability to identify patterns and relations and to infer rules for novel problems (Horn and Cattell, 1966). Gf is independent from skills relying on previously learnt knowledge, commonly referred to as crystallized intelligence, but is of great importance for academic achievement (Lynn et al., 2007; Alloway and Alloway, 2010). As reasoning ability is highly related to WM capacity, one hypothesis has been that effects of WM training will transfer to improvements in performance on reasoning tasks. This has indeed been observed in some studies (Klingberg et al., 2005; Jaeggi et al., 2008), while other studies have not found such effects (Holmes et al., 2009; Thorell et al., 2009; Bergman Nutley et al., 2011). The inconsistent findings may reflect variability in the demographic characteristics of the participants, such as age and clinical status, the tasks used to evaluate reasoning ability (Klingberg, 2010), as well as other factors associated with the training programs such as motivation (Jaeggi et al., 2011).

In addition, within the same training condition, inter-individual differences might be important for predicting training improvements and transfer. For example it has recently been reported that variants within the gene coding the dopamine transporter (*DAT1*) influence the degree of transfer following cognitive training (Söderqvist et al., 2012). Other studies have reported correlations between baseline cognitive capacity and improvements following training (Mackey et al., 2011) and between training progress and degree of transfer (Jaeggi et al., 2011). In clinical samples with large heterogeneity in both baseline capacity and etiology such inter-individual differences might be of particular importance as they might reflect on the capacity to learn and improve from practice.

Considering the difficulties of inducing transfer effects to reasoning ability following WM training, an alternative approach is to train directly on tasks that load highly on reasoning ability. One study assessed this by using commercially available games (Mackey et al., 2011). Two groups of children were compared: one group playing games considered to emphasize speeded responses and the other playing games considered to require reasoning abilities. Analysis of pre- and post-scores showed significant improvements on the functions being trained. In addition the reasoning training resulted in improved visuo-spatial WM.

We recently developed a computerized program targeting non-verbal reasoning (NVR) specifically (Bergman Nutley et al., 2011). The program was based on three tests from the Leiter test battery (Roid and Miller, 1997) all loading on Gf: Repeated Patterns, Classification, and Sequential Order. Similar to the WM training described above, an adaptive algorithm was used to ensure that training was performed at a level close to each participant's highest capacity and the training did not include any instructions regarding strategy use. This program was assessed in typically developing 4-year-old children who trained for approximately 15 min per session for a minimum of 20 sessions. Compared to an active control group, the training group showed significant improvements on a measure of Gf. Furthermore, training NVR resulted in transfer effects to a visuo-spatial WM task, demonstrating transfer between cognitive constructs.

One clinical group for which cognitive training could be of particular benefit is children with intellectual disabilities. In addition to impaired intelligence, these children often show impaired performance on both visuo-spatial and verbal WM (Van der Molen et al., 2009). Although WM is strongly correlated with Gf (Engle et al., 1999; Conway et al., 2003), these impairments are not mediated by Gf deficits as WM impairments remain after controlling for Gf (Van der Molen et al., 2009). Intellectual disability thus includes independent deficits in both Gf and WM, which suggest that children with such disabilities might benefit from interventions aimed to improve WM as well as NVR. A number of studies have attempted to improve WM in patients with intellectual disabilities. Initial studies focused on teaching rehearsal strategies and some studies did show that this approach can improve WM performance (Brown et al., 1973; Kramer and Engle, 1981; Conners et al., 2001, 2008). However, no advantage from teaching rehearsal strategies was found compared with training without specific strategy related instructions (Kramer and Engle, 1981). Recently a WM training program focusing on repeated and intense training without any rehearsal strategies was assessed in a population of intellectually impaired teenagers (Van der Molen et al., 2010). Training on a visuo-spatial WM task (an Odd One Out task) resulted in significantly improved performance on a compound measure of verbal WM (digit and non-word recall) directly after training had finished. Additional encouraging results emerged at a 10-week follow-up with significant improvements observed on visual WM and on measures of school achievement and story recall. However, this study did not yield significant improvements on Raven's progressive matrices, a reasoning task known to load highly on Gf. These findings suggest that it is possible to train visuo-spatial WM in intellectually impaired young people and, importantly, that such training can lead to improvements on non-trained WM tasks, also in the verbal domain.

The current study assessed training in children with intellectual disability using a combination of visuo-spatial WM and NVR training as previously used in typically developing children by Bergman Nutley et al. (2011). The first aim of the current study was to assess whether children with intellectual disability can manage the intense regime of cognitive training. Second, we aimed to evaluate if successful training in children with intellectual disability leads to improved performance on non-trained tasks. Considering the large heterogeneity of etiology and severity of symptoms within this group of children we expected a large variability in response to the intervention. The third aim was therefore to evaluate predictors of inter-individual differences in training progress and transfer.

## Materials and methods

### Participants

All participants had intellectual disability (IQ < 70, retrieved from clinical records) and were registered with the mental habilitation center in the area of Buskerud in Norway. Guardians of patients with intellectual disability and with a chronological age of 6–12 years were initially contacted by mail or telephone and invited to participate in the study. Informed consents were obtained from legal guardians and children before participation. Ethical approvals were received from the regional ethics committees at Oslo University and Karolinska Institutet in Stockholm. We included children aged 6–12 years, rather than older children, to ensure the program was age appropriate regarding motivational aspects. All children were pseudo-randomized into the two training groups, after controlling for chronological age and gender by independent personnel not otherwise involved with study design or implementation. The study had a double-blinded design, with participants and cognitive assessors being blind to group membership. In order to be able to generalize our results to wider clinical samples of children with intellectual disabilities, we included children with additional co-morbid diagnoses and/or taking prescribed medication. Exclusion criteria were a diagnosis of autism and severe motor and sensory problems, as these were considered to affect pre- or post assessments (and hence reliability of assessments) or training ability. For practical reasons children with guardians requiring an interpreter for conversations in Norwegian were also excluded.

### Cognitive assessments

Assessments included verbal and visuo-spatial WM tasks, measures of NVR tasks loading on Gf, sustained attention, and language functioning. All tests were administered before training (T1), directly after the training period (T2), and 1 year after the training (T3). Tests were administered in the same order at all time points. A word span task was used to assess verbal short term memory (STM) and WM (Thorell and Wahlstedt, 2006). In the STM condition, a series of non-related nouns are presented verbally to the child who is required to repeat these in the correct forward order. Each trial consists of a string of words to be remembered starting with a load of two (i.e., a string of two words to be remembered), load is then increased as the participant answers correctly, with a maximum load of six. The test ends after four consecutive incorrect answers. In the WM condition, the task is changed to include manipulation of information by requiring the participant to recall strings of words in the backwards order to their presentation but with otherwise similar procedure. To assess visuo-spatial WM we used the Odd One Out task from the Automated WM Assessment (Alloway, 2007). This computerized task requires the participant to first identify the odd shape in a series of three shapes presented simultaneously in three boxes. Three empty boxes are then presented and the child has to point to that box in which the odd shape appeared. Difficulty is increased by increasing the number of series presented sequentially, and hence how many locations one needs to remember (one location for each series presented).

Two measures loading on Gf were used: Block Design from Wechsler Preschool and Primary Scale of Intelligence (WPPSI) (Wechsler, 2004) and Raven's colored progressive matrices (Raven, 1998). The Block Design task requires the participant to reproduce a visually presented pattern using red and white colored blocks. Scores are calculated based on speed and accuracy, with a maximum score of 40. The Raven's colored progressive matrices test involves completing incomplete matrices by identifying visual patterns and rules. To reduce test-retest effects and shorten the time of assessment, we administered even numbered items of Raven's colored matrices at T1 and odd numbered items at T2 and T3. The maximum score was 18. The Auditory Attention subtest from the NEPSY (Brooks et al., 2009) was used to assess sustained attention. During 3 min the participant listens to a recorded voice pronouncing list of words read with a 1 s interval and the child has to place a red foam figure in a box each time the word “red” is heard. Points are given for each correct response and withdrawn for each incorrect response (placing a red figure in the box when the word “red” was not heard, or responding to the mentioning of some other color by placing figures with that color in the box). The Comprehension of Instructions (Instructions) subtest from the NEPSY was used to assess language comprehension. The child is instructed to point to figures with certain characteristics in the same order as instructed. Task difficulty increases with number of items, number of characteristics, and their syntactic complexity.

### School assessments

A Norwegian translation of the Aston Index test for language disabilities (Newton and Thomson, 1982) was used to assess letter reading and writing. Number perception and calculations were assessed using the Norwegian paper-and-pencil assessment “Alle Teller” (McIntosh, 2007). These were assed directly before training and 1 year following training.

### Parent-rated behavioral questionnaires

Parents completed questionnaires at T1, T2, and T3. A Norwegian translation of The Strengths and Difficulties Questionnaire (SDQ) (Heyerdahl, 2003) was used to measure child behavior on five scales: emotional symptoms, conduct problems, hyperactivity/inattention, peer relationship problems, and prosocial behavior. A revised version of the diagnostic questions for ADHD from the DSM-IV (American Psychiatric and American Psychiatric Association, 2000) were used to assess inattention.

### Motivation

To assess children's motivation for performing the training programs we asked the children's parents (or teachers when the training was carried out at school) to complete an in-house questionnaire with eight questions on a 5 point scale. Questions concerned how fun, entertaining, and difficult the training was perceived by the parent/teacher and how the parent/teacher believed that the child had perceived the training.

### Training procedure

Training was carried out in either the participants' home with parent supervision (80% of participants) or at school with teacher supervision. Training was performed for approximately 20 min a day, 5 days a week for 5 weeks using participants' or schools' personal computers. A minimum of 20 training sessions were required for inclusion in analyses. At each training session the participants trained on two (out of three) different versions of the NVR tasks and two (out of seven) different versions of the WM tasks. The NVR tasks consisted of a display of different cards with different geometrical shapes that could be altered in a number of different parameters (e.g., color, shape, size). For each task one or two slots were empty and the participants had to allocate cards from a set of alternatives to fill these slots. The three different types of tasks were: *Repeated Patterns* that required the completion of a repeated pattern such as alternating shapes; *Sequential Order* in which a logical progression (e.g., increase in size) had to be identified to complete the pattern; and *Classification*, which required the matching of target cards to the correct alternative that matched on some parameter, such as the same color (for a more detailed description of the training paradigms see Bergman Nutley et al., 2011). The WM training program was provided by Cogmed Systems and consisted of visuo-spatial WM tasks. Colorful figures were displayed in different settings (e.g., in a pool or riding on a roller-coaster) and some of the figures made sounds (e.g., laughing) and changed color in a serial order. The task was to click on the figures in the same order as they had made a sound and changed color. The number of figures to be remembered was increased for each level. Difficulty level was automatically adjusted according to performance in the adaptive training group, but was always kept at the lowest level (one item to be remembered) in the non-adaptive training group.

Training performance was monitored by researchers via an internet server for both training groups to ensure that training was being performed and that each session lasted approximately 20 min. Furthermore, performance for the adaptive training group was monitored to assess improvements. Feedback was provided to all participants individually via e-mail once a week.

### Statistical analyses

To test the effect of training we performed univariate general linear models in SPSS (version 20.0.0) using each of the outcome measures as a dependent variable and including T1 performance on the same measure, age, gender, group, and a group^*^gender interaction as independent variables. In order to account for differences in training progress and how these affect transfer, further analyses using training improvement as a continuous independent variable were performed. For the three NVR tasks we used scores of the highest levels reached on the different tasks. For the non-adaptive training group this was set to three which was the highest level their training could reach. For performance on WM training tasks we used an index improvement score based on the highest level reached, but taking into account baseline performance, measured as the performance during the second and third day of training when it is assumed that no training improvements have yet occurred. Participants in the non-adaptive training group were constantly on level one throughout the training and their index improvement was set to zero. These measures were all standardized and a mean score of these standardized scores was used to represent each participant's training progress. This measure of training progress was later also used as a dependent variable in backwards stepwise regression analyses assessing how baseline performance predicted training progress in the adaptive training group.

## Results

### Demographics

Out of 52 participants recruited, 41 were included in the analyses (22 males and 19 females), aged 6–12.5 years (*M* = 9.68, SD = 1.58). Children were excluded due to problems with T1 assessments (e.g., poor engagement in tasks) (*n* = 3), not completing the required 20 sessions of training (*n* = 7) and technical problems causing incomplete training data (*n* = 1). Twenty-two children were included in the adaptive training group and 19 children were included in the non-adaptive training group. Training was performed for 20–25 sessions (*M* = 24.5, SD = 1.50 in the adaptive training group and *M* = 24.7, SD = 1.16 in the non-adaptive training group).

According to parental reports, 20 participants had additional diagnoses: 9 with ADHD (non-adaptive training *n* = 4, the adaptive training *n* = 5), 2 with Down's syndrome (non-adaptive training *n* = 1, adaptive training *n* = 1), 2 with epilepsy (non-adaptive training *n* = 1, adaptive training *n* = 1), and 7 with other additional neurological diagnoses: 1 with Albrik's syndrome (adaptive training), 2 with unspecified chromosomal deviation (non-adaptive training *n* = 1, adaptive training *n* = 1), 1 with language disorder (adaptive training), 1 with Duchenne muscular dystrophy (adaptive training), 1 with Hypothalamic insufficiency (non-adaptive training), and 1 with neurofibromatosis-1 (adaptive training). Five participants were prescribed psycho stimulant medication throughout the study period (non-adaptive training *n* = 2, adaptive training *n* = 3).

*T*-tests revealed no significant differences in baseline performance or age between the two groups (all *p*-values >0.1) (Table 1 summarize performance across groups and time-points). Similarly, Chi Square tests showed no significant differences in the distribution of gender and number of co-morbid diagnoses between the two training groups (both *p*-values >0.1). *T*-tests comparing baseline performance for the two genders showed a trend effect of males performing better than females on word span backwards [*t*_(38)_ = 1.85, *p* = 0.072]. Due to this observation we included gender as a covariate in all subsequent analyses.

**Table 1:** **Mean scores for the two training groups at the three assessment points**.

	**Adaptive training group**			**Non-adaptive training**				
	**T1 Mean (SD)**	**T2 Mean (SD)**	**T3 Mean (SD)**	**T1 Mean (SD)**	**T2 Mean (SD)**	**T3 Mean (SD)**	**T2 Cohen's *d***	**T3 Cohen's *d***
Word span backwards	5.48 (5.29)	7.10 (6.93)	6.71 (8.19)	6.25 (7.50)	5.31 (4.80)	7.94 (8.37)	0.41	−0.07
Word span forwards	14.76 (4.62)	13.33 (5.16)	13.38 (6.64)	11.63 (5.95)	13.88 (6.35)	13.69 (6.85)	−0.15	−0.37
Odd One Out	9.59 (4.30)	11.45 (5.21)	11.09 (5.42)	10.31 (4.47)	10.38 (4.41)	11.88 (5.58)	0.40	−0.02
Block Design total	24.27 (4.23)	25.09 (5.04)	24.18 (5.12)	22.81 (4.40)	22.50 (4.76)	23.38 (6.61)	0.27	−0.15
Block Design females	25.40 (3.53)	23.80 (5.03)	24.20 (4.85)	22.14 (2.73)	20.86 (2.27)	21.29 (6.08)	−0.09	−0.1
Block Design males	23.33 (4.68)	26.17 (5.01)	24.17 (5.56)	23.33 (5.48)	23.78 (5.87)	25.00 (6.89)	0.10	−0.04
Instructions total	14.70 (4.98)	16.20 (4.65)	16.10 (4.79)	14.06 (4.80)	15.12 (4.96)	16.18 (4.73)	0.09	−0.15
Instructions females	15.25 (2.77)	17.27 (3.41)	16.50 (3.30)	13.43 (5.26)	13.14 (4.74)	16.00 (6.11)	0.55	−0.32
Instructions males	14.33 (6.13)	15.50 (5.35)	15.83 (5.70)	14.50 (4.70)	16.50 (4.86)	16.30 (3.86)	−0.16	−0.06
Auditory Attention	37.62 (22.03)	43.67 (21.89)	46.29 (18.94)	37.46 (20.03)	40.85 (22.38)	45.92 (16.66)	0.11	0.01
Raven's	8.95 (3.87)	8.15 (3.30)	8.55 (2.91)	8.00 (4.20)	7.25 (3.44)	8.19 (2.83)	−0.01	−0.15

Effect sizes of adaptive training compared to non-adaptive training are represented by Cohen's d for change from T1 at T2 and at T3. For the two tests showing gender interactions, scores are also presented for the two genders separated.

### Motivation

Parents responded to statements about their perceptions of the training. The adaptive training group agreed to a larger extent with the statement that the training was too difficult [χ^2^_(4)_ = 16.50, *p* < 0.05], while the non-adaptive training group agreed to a larger degree with the statement that training was too easy [χ^2^_(4)_ = 14.99, *p* < 0.05], as measured with Pearson's chi-square test. However there were no significant differences between the training groups on questions regarding how entertaining or motivating the training was perceived (all *p*-values >0.1). Furthermore, correlating training progress within the adaptive training group revealed no significant correlations between any of the motivation parameters and training performance (all *p*-values >0.1).

### Effects of training at T2

Univariate general linear models were performed separately for the different outcome measures. Test performance at T2 was the dependent variable, gender, and training group were entered as factors and test performance at T1, age, and gender^*^training group interaction were included as covariates. Training group showed no significant effects in predicting transfer effects (word span forwards, *p* = 0.960; word span backwards, *p* = 0.104; Odd One Out, *p* = 0.107; Instructions, *p* = 0.349; Block Design, *p* = 0.387; Raven's, *p* = 0.669; Auditory Attention, *p* = 0.107). However a trend effect for the group^*^gender interaction was observed for the Instructions task [*F*_(1, 33)_ = 3.998, *p* = 0.054]. Significant effect of training group on the Instructions task was seen for females only [*F*_(1, 13)_ = 29.49, *p* = 0.049; compared to *F*_(1, 18)_ = 4.88, *p* = 0.434 for males].

### Training progress

**Figure 1:**
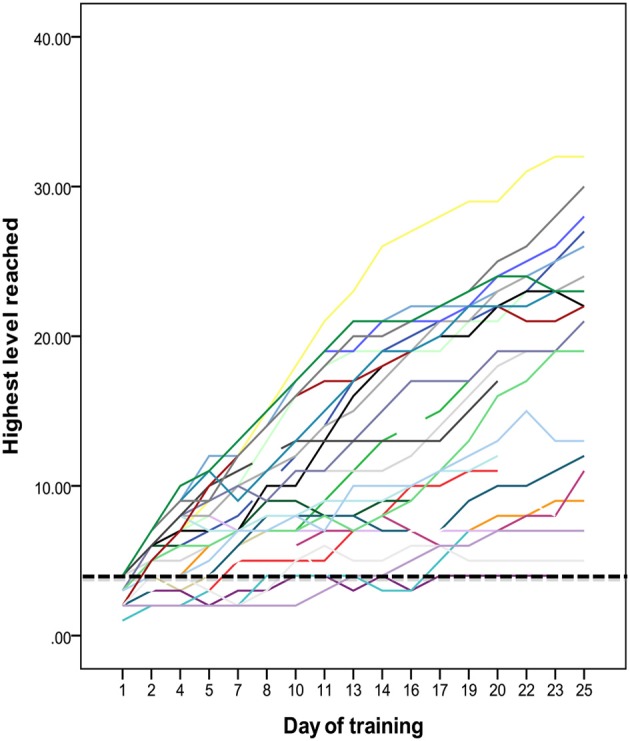
**Improvements during training on non-verbal reasoning tasks.** Each line represents one participant. Highest level of performance on each training day is shown on the *y*-axis and the *x*-axis shows the training session. The dashed line indicates the highest level performed by the non-adaptive training group throughout the training period.

There was large inter-individual variance in training progress within the adaptive training group (Figure 1). For some participants performance did not increase considerably above the levels of the non-adaptive training paradigm and for these children the training cannot be considered successful. In order to assess how differences in training progress affected transfer effects we carried out additional analyses using training progress as described above as a covariate instead of training group. General linear models were run for each outcome measure. T2 performance on each outcome measure were the dependent variables, and independent variables were T1 performance, age, gender, training progress, and a gender^*^training progress interaction. Table 2 summarizes these results. Training progress predicted improvements on Odd One Out [*F*_(1, 34)_ = 6.53, *p* = 0.015] and word span backwards [*F*_(1, 33)_ = 7.58, *p* = 0.010]. For Comprehension of Instructions there was a significant effect of the gender^*^training progress interaction [*F*_(1, 33)_ = 4.76, *p* = 0.036], with significant effect of training progress observed for female participants only [*F*_(1, 13)_ = 5.41, *p* = 0.037; compared to *F*_(1, 18)_ = 0.77, *p* = 0.391 for males]. For Block Design we observed a trend for training^*^gender interaction [*F*_(1, 33)_ = 3.33, *p* = 0.077]. Effects of training were associated with improvements on Block Design in males with a trend effect [*F*_(1,17)_ = 13.48, *p* = 0.062], which was not observed in females [*F*_(1, 14)_ = 0.30, *p* = 0.595]. No significant effects of training progress were observed for improvements on word span forwards, Raven's colored matrices or for Auditory Attention (all *p*-values >0.1). For measures of WM, the analyses of training progress explained transfer improvements to a greater extent compared to the training group analyses. These results show that larger improvements during training were associated with greater training gains.

**Table 2:** **The effect of training progress on transfer effects**.

**Outcome measure**	**R^2^**	**T1 performance *F* (*p*)**	**Age *F* (*p*)**	**Gender *F* (*p*)**	**Training progress *F* (*p*)**	Training progress^*^ gender *F* (*p*)
Word span backwards	0.56	**33.06 (<0.001)**	0.04 (0.837)	0.27 (0.607)	**7.58 (0.010)**	1.03 (0.317)
Word span forwards	0.47	**29.44 (<0.001)**	0.02 (0.904)	0.00 (0.961)	0.13 (0.718)	0.00 (0.981)
Odd One Out	0.69	**58.23 (<0.001)**	0.46 (0.504)	1.83 (0.185)	**6.53 (0.015)**	0.019 (0.892)
Block Design	0.56	**28.37 (<0.001)**	0.67 (0.420)	**7.22 (0.011)**	1.16 (0.289)	3.33 (0.077)
Raven's colored matrices	0.46	**6.56 (0.015)**	2.88 (0.099)	3.44 (0.072)	0.83 (0.369)	0.205 (0.654)
Comprehension of instructions	0.71	**50.51 (<0.001)**	0.44 (0.511)	1.19 (0.283)	0.717 (0.403)	**4.76 (0.036)**
Auditory Attention	0.76	**50.57 (<0.001)**	0.05 (0.833)	0.01 (0.923)	0.11 (0.744)	1.38 (0.249)

Table shows F and p-value for the factors and covariates included in the analysis of each outcome measure: T1 performance on the outcome measure, age, gender, training progress, and training progress

* gender interaction. Adjusted R^2^ for each model is also presented. Significant values (p < 0.05) are marked in bold.

### Effects of training at T3

Training had no effect on outcome measures employed in this study assessing cognitive abilities or school assessments at the T3 follow-up at the group level. There were also no strong relationships between progress during training and performance at T3 (all *p*-values >0.1).

### Parent-rated behavioral questionnaires

No significant training related changes were observed in scores on the ADHD symptoms and the Strength and Difficulties questionnaires at T2 or T3 (all *p*-values >0.1).

### Prediction of training progress

To investigate predictors of training progress we performed backwards stepwise regression analysis including participants from the adaptive training group only. We included all cognitive measures at T1, gender, and co-morbid diagnosis as a categorical variable (yes/no) as independent variables and training progress as the dependent variable. The final model with best prediction of training progress included 5 variables: gender (β = 0.573, *p* = 0.001); backwards word span (β = 0.516, *p* = 0.003); co-morbidity (β = −0.513, *p* = 0.002); word span forwards (β = 0.315, *p* = 0.069); and Block Design (β = −0.294, *p* = 0.071). These results show that females and participants with an intellectual disability but no additional diagnosis on average had more progress during training. On cognitive tasks, high performance on the backwards and forward word span tasks was associated with greater training progress. In contrast, performance on the Block Design task was negatively associated with progress, with lower baseline performance associated with greater training progress.

## Discussion

The major finding of this study is that it is feasible for children with intellectual disability to undergo intensive computerized cognitive training, with more than 85% of participants completing approximately 20 min of training per session for an average of 24 (and minimum of 20) sessions. There was large variability in training performance with some participants showing little progress during training. The amount of progress during training was significantly related to improvements on transfer tasks measuring visuo-spatial and verbal WM and language comprehension. Training progress predicted improvements on both WM and language comprehension directly following training, but not at a 1-year follow-up. Training on purely visuo-spatial tasks resulted in improvements tasks assessing verbal WM and language function, thus showing transfer between cognitive constructs and modalities. This is particularly encouraging as deficits in verbal WM are observed to be more severe than visuo-spatial deficits in children with intellectual disabilities (Henry and MacLean, 2002; Van der Molen et al., 2009).

**Figure 2:**
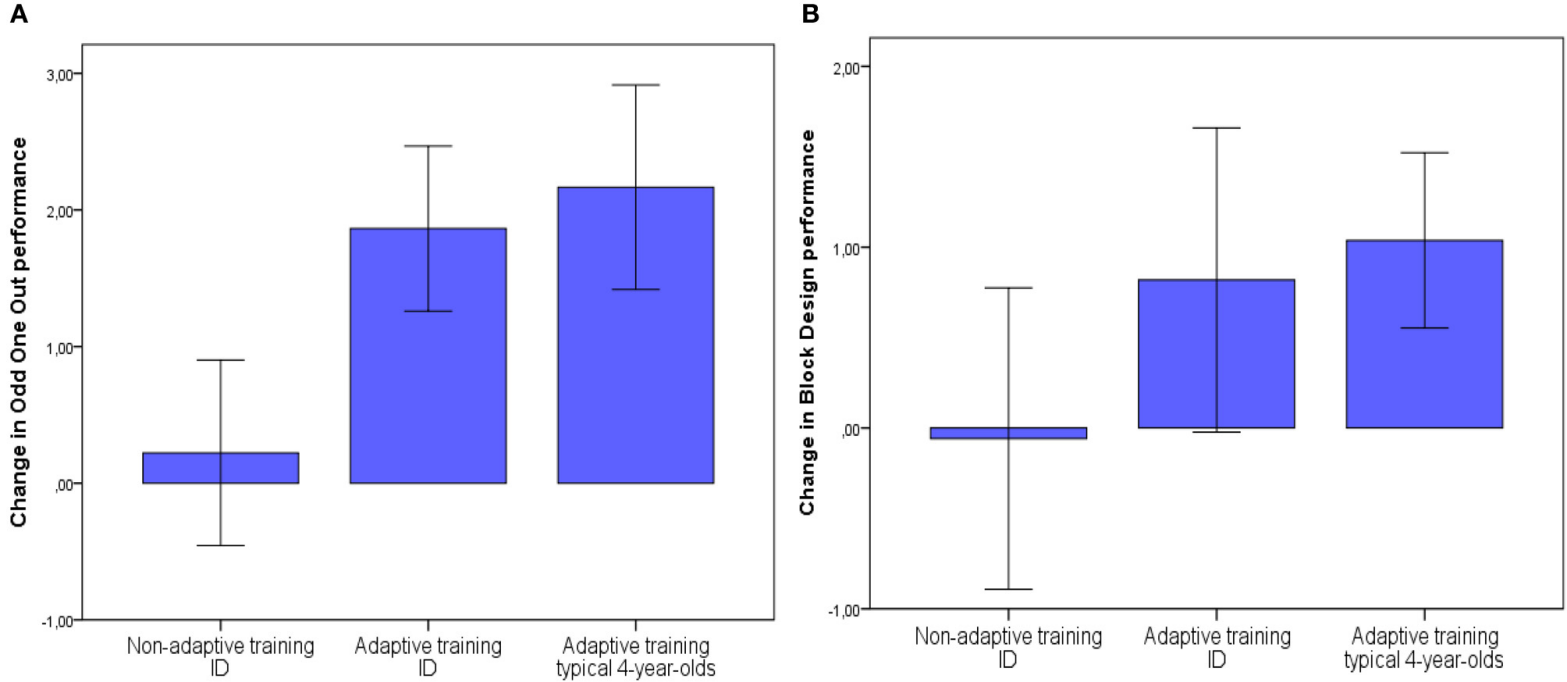
**Mean improvements following training (T2—T1) on Odd One Out (A) and Block Design (B) for the two training groups of children with intellectual disability (ID) and the adaptive (combination) training group of typically developing 4-year-olds as reported in Bergman Nutley et al., 2011.** Error bars show +/− 1 standard error of the mean.

Training did not lead to significant improvements on reasoning ability tasks (Block Design and Raven's colored matrices) although a trend association was observed on improvements on Block Design for males. Figure 2 shows improvements on a WM task (Odd One Out) and a reasoning task (Block Design) for the two groups in the current study as well as for the typically developing sample of 4-year-olds who previously completed the same training (reported in Bergman Nutley et al., 2011). As is apparent from this figure, adaptive training resulted in similar improvements in WM for the children with intellectual disability as it did for the typically developing 4-year-olds. However, in the current sample improvements on Block Design were of a smaller magnitude and with larger variability compared to the typically developing sample. This suggests that reasoning ability is more difficult to improve with training in this clinical group, perhaps due to this deficit being particularly impaired in children with intellectual disability.

The importance of training progress for transfer has recently also been demonstrated by Jaeggi et al. (2011), who showed that transfer effects following WM training were dependent on improvements observed during training in typically developing children. However, no significant relation between baseline capacity and training performance was found, thus failing to explain what determined successful training for the participants. This emphasizes the importance of studying inter-individual differences in how cognitive training is received, which has been overlooked in the majority of previous training studies. Increased understanding of this can be of great importance for guiding the future development of cognitive training programs and practices. It might be of particular importance in clinical groups that show large heterogeneity in etiology and baseline capacity, as examined in the current study.

In the clinical group currently studied, performance on the verbal WM task at baseline together with co-morbid diagnosis and gender were the strongest predictors of training progress, suggesting that verbal WM is of particular importance. Considering the evidence that verbal WM is specifically impaired in populations with intellectual disabilities (Van der Molen et al., 2009), performance on the verbal WM task might be an indication of severity of impairment, which in turn might affect the susceptibility to training induced plasticity. In general we observed that high performance at baseline was associated with larger progress during training and a higher level of transfer effects. Similar findings were found by Conners et al. (2008) for a verbal rehearsal task in children with Down's syndrome.

One possible explanation for the lack of progress for participants with low baseline scores could be that baseline capacity for these children falls under some threshold required to perform the tasks in the program. In order to assess this we compared baseline performance, on study-overlapping tasks, with that of the typically developing 4-year-olds participating in the Bergman Nutley et al. (2011) study, who did show transfer effects. We found that, at baseline, participants in the current study performed equally well or significantly higher on measures of visuo-spatial WM (Odd One Out) and on measures of fluid intelligence (Block Design and Raven's colored matrices). This implies that the problem for the low performing group in this study is not related to their low baseline capacity *per se*. Rather, it is suggested that their relative low baseline capacity reflects a reduced level of plasticity that leads to smaller effects of transfer compared to that observed for the typically developing 4-year-olds. Perhaps participants with low level of plasticity require alternative methods of training, such as changed length of training period or changes in the adaptive algorithm that would allow a slower progress and therefore more practice on each level. It may also be beneficial to focus training on one construct (WM or NVR) at a time, allowing for more time being spent training on either one. This is supported by previous findings that amount of transfer seems to follow linearly from amount of time spent training that construct (Bergman Nutley et al., 2011). These issues are for future studies to investigate.

Furthermore, whether the predictive power of high baseline capacity relating to greater progress during training and larger transfer effects is special for clinical populations like this or can be generalized to healthy populations requires more in-depth investigations as some studies suggest the opposite pattern. For example, Mackey et al. (2011) found that typically developing children with lower Gf scores at baseline gained more from training than those starting with higher Gf scores. One possible explanation is that the association with poorer performance on baseline measures and larger gains in Gf reflects a regression toward the mean effect; that is, children who by chance perform below their optimal level at baseline (due to uncontrolled confounders such as energy levels, motivation, and current health status) are more likely to perform closer to their optimal level at the follow-up assessments. We take this into consideration in the current study by controlling for baseline performance in our analyses.

A concern when interpreting our results is whether the larger transfer effects we see for high performing individuals are in fact a result of the training related improvements, or whether these effects reflect a general higher level of plasticity in the high performing group, resulting in higher test-retest effects. If the latter was the case we would also expect there to be a positive correlation between baseline performance and improvements on T2 measures in the non-adaptive training group. This was not observed; rather as would be expected with a regression toward the mean effect, all significant correlations were negative indicating that lower performance on T1 measures was associated with higher gains in performance on T2 measures.

Further investigation is needed to better understand the role of co-morbid diagnoses and gender. It is at the moment not clear to us why gender would have such a strong influence in predicting training effects as we observed here, and these findings need further replication and investigation. Other factors that we were not able to control for in this study but are likely to influence training effects are underlying etiology and genetic variability.

We did not observe significant training effects at the 1-year follow-up. This suggests that training in children with intellectual disability needs to be more extended (e.g., 10 weeks instead of 5) or repeated (e.g., 5 weeks every 3 months) in order for effects to be maintained. It is not clear what frequency and intensity would be required or whether this is specific for children with intellectual disability or would also generalize to other clinical and non-clinical groups of children.

In summary, we provide new encouraging evidence that cognitive functions can be trained and improved in some children with intellectual disability. We also highlight the importance of looking at inter-individual differences in training performance and show that these predict transfer effects resulting from the training. Understanding who benefits from which type of training can help in developing future training programs to be better adapted to different individual capacities.

### Conflict of interest statement

Sissela B. Nutley and Torkel Klingberg are co-applicants on an international patent application for the non-verbal reasoning program, which has been financed by Pearson Assessment. Sissela B. Nutley has an employment at Pearson Assessment, who distributes the working memory training program. The other authors declare that the research was conducted in the absence of any commercial or financial relationships that could be construed as a potential conflict of interest.
